# Sorting things out: Assessing effects of unequal specimen biomass on DNA metabarcoding

**DOI:** 10.1002/ece3.3192

**Published:** 2017-07-28

**Authors:** Vasco Elbrecht, Bianca Peinert, Florian Leese

**Affiliations:** ^1^ Aquatic Ecosystem Research Faculty of Biology University of Duisburg‐Essen Essen Germany; ^2^ Centre for Water and Environmental Research (ZWU) Essen University of Duisburg‐Essen Essen Germany

**Keywords:** biomass bias, DNA barcoding, ecosystem assessment, metabarcoding, metagenomics, size sorting

## Abstract

Environmental bulk samples often contain many different taxa that vary several orders of magnitude in biomass. This can be problematic in DNA metabarcoding and metagenomic high‐throughput sequencing approaches, as large specimens contribute disproportionately high amounts of DNA template. Thus, a few specimens of high biomass will dominate the dataset, potentially leading to smaller specimens remaining undetected. Sorting of samples by specimen size (as a proxy for biomass) and balancing the amounts of tissue used per size fraction should improve detection rates, but this approach has not been systematically tested. Here, we explored the effects of size sorting on taxa detection using two freshwater macroinvertebrate bulk samples, collected from a low‐mountain stream in Germany. Specimens were morphologically identified and sorted into three size classes (body size < 2.5 × 5, 5 × 10, and up to 10 × 20 mm). Tissue powder from each size category was extracted individually and pooled based on tissue weight to simulate samples that were not sorted by biomass (“Unsorted”). Additionally, size fractions were pooled so that each specimen contributed approximately equal amounts of biomass (“Sorted”). Mock samples were amplified using four different DNA metabarcoding primer sets targeting the Cytochrome c oxidase I (COI) gene. Sorting taxa by size and pooling them proportionately according to their abundance lead to a more equal amplification of taxa compared to the processing of complete samples without sorting. The sorted samples recovered 30% more taxa than the unsorted samples at the same sequencing depth. Our results imply that sequencing depth can be decreased approximately fivefold when sorting the samples into three size classes and pooling by specimen abundance. Even coarse size sorting can substantially improve taxa detection using DNA metabarcoding. While high‐throughput sequencing will become more accessible and cheaper within the next years, sorting bulk samples by specimen biomass or size is a simple yet efficient method to reduce current sequencing costs.

## 1 INTRODUCTION

Recent advancements in high‐throughput sequencing (HTS) and DNA barcoding have improved our ability to rapidly assess biodiversity. Using traps or manual collection methods (e.g. nets), thousands of specimens can be easily collected. However, manually identifying hundreds or thousands of specimens in a single sample is often not feasible, especially if species level identification is needed (Haase et al., 2004). Bulk samples, which previously took weeks or months to determine morphologically, can now be homogenized and their DNA extracted for sequencing based identification within days. The power, accuracy, and cost‐effectiveness of these HTS based assessments have already been demonstrated (e.g., Ji et al., 2013; Tang et al., 2015; Gómez‐Rodríguez et al., 2015; Leray & Knowlton, 2015; Gibson et al., 2014; Hajibabaei et al., 2011; Zimmermann et al., 2014; Dowle, Pochon, & Banks, 2015; Elbrecht et al. 2017), and sequencing costs are expected to further decline in the future.

In DNA‐based ecosystem assessment, we can distinguish between two approaches: (1) A target gene fragment is amplified and compared to a DNA barcoding database (metabarcoding, see Taberlet et al., 2012), or (2) the extracted DNA from the bulk sample is shotgun sequenced directly without PCR and can be optionally enriched for target genes (metagenomics, see Liu et al., 2016; Crampton‐Platt et al., 2016). Both approaches have specific advantages and drawbacks: Metabarcoding is severely limited by PCR bias, preventing estimates of taxa biomass and potentially not detecting all taxa present in the sample (Elbrecht & Leese, 2015; Leray & Knowlton, 2015; Piñol et al., 2014). While metagenomics might overcome these PCR‐based problems, this approach is currently limited because only little reference data is available (e.g., mitochondrial genomes), and a high sequencing depth is required (Crampton‐Platt et al., 2016). Additionally, both approaches are likely affected by variable cell densities and types, as well as variable mitochondrial genome copy numbers between taxa and specimen life stages (Ballard & Whitlock, 2004; Moraes, 2001), which is potentially affecting taxa detection. While these problems might be solved at least partially by optimized degenerate primers (Elbrecht & Leese, 2017), reduced sequencing costs and mitogenome capture (Tang et al., 2014), both metabarcoding and metagenomics approaches are potentially affected by an additional factor: variable taxa biomass.

Environmental samples usually contain a diverse set of taxa spanning often several orders of magnitude in specimen size and consequently biomass. When extracting complete bulk samples, large biomass rich specimens will contribute significantly more DNA to the final bulk DNA isolate than small organisms with little biomass. We demonstrated this previously, by bulk extracting DNA from 31 specimens of the same stonefly (Plecoptera) species with varying specimen biomass, and found a clear significant linear correlation between obtained reads and dry specimen weight (*p* < .001, *R* ^2^ = .65, Elbrecht & Leese, 2015). We hypothesize that also in more species rich samples, taxa biomass translates directly into read abundance (assuming no primer bias among species, Elbrecht & Leese, 2015). Thus, just a few big specimens in a sample will likely make up the majority of the reads, requiring higher sequencing depth to also detect small specimens and rare taxa. The effects of large specimens might be also further influenced by primer bias increasing or decreasing the number of reads obtained for a taxon (Elbrecht & Leese, 2015; Piñol et al., 2014). Some DNA metabarcoding studies have already sorted samples into different size fractions, because of this biomass introduced bias (Leray & Knowlton, 2015; Wangensteen & Turon, 2016). However, the effect of fractioning samples by specimen biomass compared against processing complete sample without presorting of specimens has not yet been systematically tested and quantified. Morinière et al., 2016 detected additional taxa when sorting malaise trap samples by insect orders that sometimes have different biomass. The effects, however, could have been also caused by unequal sequencing depth between the samples. Thus authors further encourage to also test the effects of fractioning samples by specimen biomass instead of orders.

**Figure 1:**
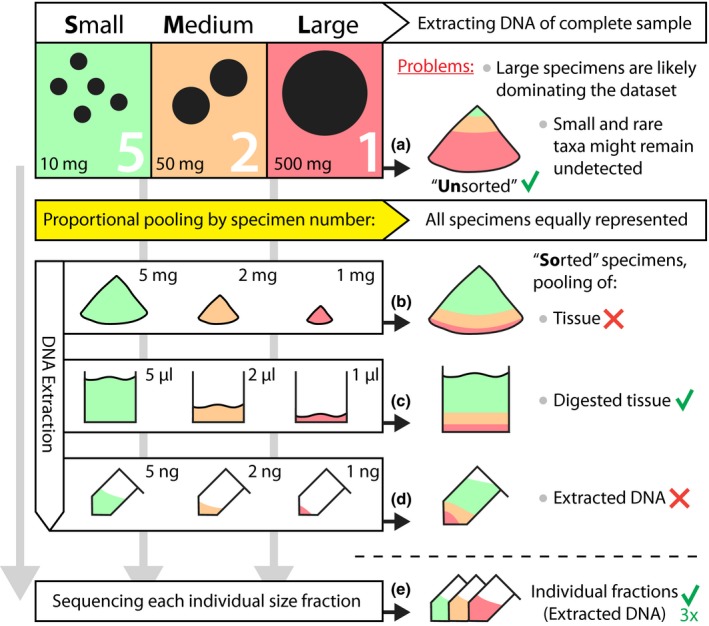
Overview of different strategies to reduce the presence of biomass rich specimens when metabarcoding bulk samples. Aliquots with a green checkmark (✓) were generated and metabarcoded in this study, while those with a red “X” were not tested. Large specimens (L) have substantially more biomass than small specimens (S) and thus contribute more DNA when extracting complete *unsorted* samples (a). This likely leads to metabarcoding datasets being dominated by a few biomass rich or abundant taxa, while small and rare ones might remain undetected. If the goal of the study is to detect all taxa present in the sample it might make sense to adjust the biomass to have all specimens equally strong represented in the dataset. This can be performed by *sorting* specimens into size categories (e.g., small, medium, and large specimens), followed by sequencing of individual size fractions (e) or pooling them proportionally based on specimen abundance in each fraction (see b, c and d). It is, however, difficult to precisely pool ground tissue (b). Extracted DNA on the other hand has to be quantified and might be affected by copy number variation of mitochondrial genomes between taxa (d). Thus, in this study pooled digested tissue from each size category (c) was used to investigate the effects of sorted and unsorted samples

In this study, we systematically quantified the effects of biomass sorting on taxon recovery using two complete stream macroinvertebrate kick samples (mostly larval specimens). Specimens of both samples were morphologically identified and sorted into three biomass categories based on specimen sizes: small (S), medium (M) and large (L), see Figs 1a and S1. These size fractions were used to generate mock samples to compare the effect of extracting all specimens together without sorting (“Unsorted”) against pooling the sorted samples according to number specimens in each sample (“Sorted”), to archive a more equal representation of all specimens in the extracted sample (Fig. 1). While it is difficult to accurately pool the ground tissue of each size category (Fig. 1b), pooling extracted DNA might be potentially biased by variable cell sizes and mitochondrial copy numbers in different taxa (Bendich, 1987; Lemire, 2005) and requires quantification (Fig. 1d). Thus, we decided to pool the DNA extraction buffer after tissue digestion of S, M, and L size fraction for mock sample generation, as the lysis buffer has the same DNA proportions as the ground tissue but can be more precisely pooled (by pipetting, see Fig. 1c). Additionally, DNA from each size category was extracted and sequenced individually, to estimate which taxa are present in each and are thus expected to be also detected in the mock samples. By metabarcoding these individual size fractions as well unsorted and sorted samples mock samples, we can precisely investigate the effects of sample sorting by specimen size on taxa recovery.

## 2 MATERIAL AND METHODS

Figure S2 gives an overview of how samples were collected, extracted, pooled into mock communities and metabarcoded as will be discussed in the following.

### 2.1 Sample collection and processing

Macroinvertebrates were collected at two sampling points of the small low‐mountain range stream Kleine Schmalenau in Germany (Arnsberger Wald). The main stream (site P8, N51.43623 E8.13721) and a small tributary (site P10, N51.43295 E8.14350) were sampled with five kick samples per sampling site (0.45 m^2^ area) following the general principle of the multihabitat sampling protocol also used in the German implementation of the EU Water Framework Directive (Meier et al., 2006). Collected specimens were stored in 96% ethanol at −20°C for later molecular analysis. All invertebrates were counted and identified morphologically to the lowest taxonomic level that could be accurately and consistently determined given the available literature, larval life stage, and specimen condition (Table S1).

Specimens from the two samples were each sorted into three size categories under a Zeiss Stemi 2000 stereo microscope by placing them onto millimeter paper (Fig. S1c). Specimens below 2.5 × 5 mm body size (length x height, excluding thin extremities and appendices) were sorted into small (S) specimens up to 5 × 10 mm into medium (M) and everything bigger than that into large specimens (L, max 10 × 20 mm, see Fig. S1c). For thin but long specimens like, for example chironomids (nonbiting midges), the specimen shape was considered and evaluated if it would fit into the surface of the respective rectangle (e.g., all chironomids were sorted into the small size category despite being some times longer than 5 mm). Antennae and cerci were not counted in the measurement of body length. Goal of the sorting by specimen size was to visually separate the specimens into size categories as a proxy for biomass (see Fig. S1), as accurate measurements on ethanol wet specimens are difficult. Terrestrial taxa and Trichoptera (caddisfly) quivers were included in the samples, as it is not realistic to remove nontarget organisms or empty shells and quivers in large scale routine monitoring samples. This means that besides tissue also acellular material was part of the ground tissue power.

### 2.2 DNA extraction and tissue pooling

**Figure 2:**
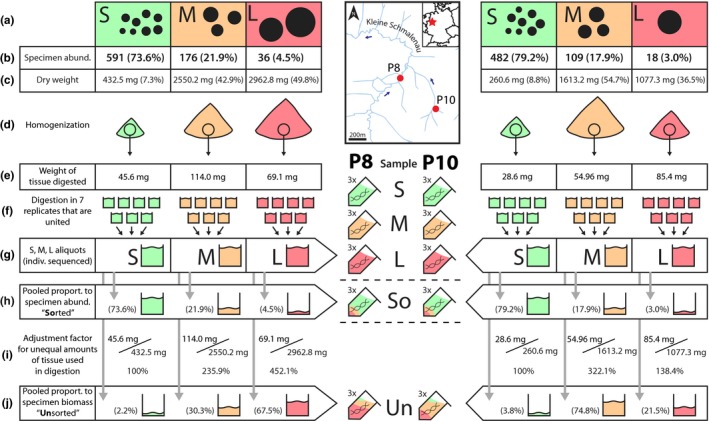
Strategies how digested tissue was pooled, to generate samples which retained the original proportion of small, medium and large specimens, as if the sample has not been sorted (“Unsorted” [j]) and a sample where size sorting did take place and specimens of each size category are proportionally pooled by specimen abundance (“Sorted” [h]). Specimens of both kick samples were sorted by specimen size into three size categories; small, medium, and large [a]. Using the specimen abundance in each category [b], as well as total dry weight [c], “sorted” and “unsorted” samples were generated by pooling digested tissue [g]) in specific proportions. To generate a unsorted mock sample digested liquid was pooled based on dry specimen weight [c] in each size category [j] under consideration of how much tissue was used in the digestion [e,i]. To adjust for specimen biomass, the sorted specimens were pooled according to the number of specimens in each size category [h]. For the sorted mock sample [h], we mistakenly did not consider the tissue adjustment factor [i]. After pooling of digested tissue, three aliquots were extracted for each category (small, medium and large specimens as well as sorted and unsorted mock samples), which each were united into a single DNA aliquot used for metabarcoding

Specimens of each size category were dried overnight at room temperature in sterile Petri dishes to remove the ethanol. Total dry specimen weight in each size category was measured (in duplicates) on a Sartorius RC 210D scale. Specimens from each category were homogenized (Fig. 2d) using an IKA ULTRA‐TURRAX Tube Drive control system with sterile 20‐ml tubes and 10 steel beads (5 mm Ø) by grinding at 4,000 rpm for 30 min (IKA, Staufen im Breisgau, Germany).

In this study, we wanted to compare the taxa recovery between samples **so**rted by specimen size and then proportionally pooled by specimen abundance (So) against **un**sorted complete samples (Un). Thus, five different DNA extractions were prepared for each of the two sampling sites (Fig. 2). First of all, DNA from each size category (S, M and L) was separately digested using a modified salt extraction protocol (Sunnucks & Hales, 1996; see Fig. S3). Seven tissue aliquots were digested and united per size category (Fig. 2f), to obtain sufficient amounts of digested tissue for pooling (Fig. 2g). Then three aliquots of digested tissue were then used to generate the sorted and unsorted mock samples. Tissue digested in DNA extraction buffer was used, as it can be precisely pooled in specific proportions (unlike ground tissue), while not introducing biases based on variation in cell density and mitochondrial copy numbers which possibly affect extracted DNA (Fig. 1). However, the amount of tissue used in digestion of S, M, and L samples was not always similar (Fig. 2e), which has to be accounted for when pooling the digested tissue for mock community generation (Fig. 2i). This, however, was mistakenly not performed for the sorted samples (Fig. 2h), where digested tissue was pooled based on the number of specimens in each size category to reduce the influence of large specimens in the extraction. This mock sample was compared with an unsorted sample pooled based on specimen weight (Fig. 2j) that retains the original tissue proportions in the sample, representing bulk DNA extraction of the complete sample. Additionally, all S, M, and L aliquots were extracted separately and used as individual metabarcoding samples, to be included as positive controls (Fig. 2). All extractions from the digested tissue were carried out in triplicates and united into one single aliquot, to increase the amount of DNA available for each sample.

Forty five microliter DNA from each sample (S, M, L, Un, So for sampling site P8 and P10) was digested with 1 μl RNAse A (10 mg/ml, Thermo Fisher Scientific, Waltham, MA, USA) and cleaned up using a MinElute Reaction Cleanup Kit (Qiagen, Venlo, the Netherlands) with resuspension in ddH_2_O. DNA concentrations were quantified fluorometrically using a Qubit (HS Kit, Thermo Fisher Scientific) and concentrations adjusted to 25 ng/μl.

### 2.3 DNA metabarcoding and bioinformatics

All 10 samples (S, M, L, Un, So for sampling sites P8 and P10) were amplified with the four freshwater macroinvertebrate fusion primer sets BF/BR (Elbrecht & Leese, 2017). The four primer combinations are targeting a 217‐ to 421‐bp long fragment of the Cytochrome c oxidase I (COI) gene. Figure S4 gives an overview of sample tagging using fusion primers with inline barcodes. Each PCR was composed of 1× PCR buffer (including 2.5 mmol/L Mg^2+^), 0.2 mmol/L dNTPs (Thermo Fisher Scientific), 0.5 μmol/L of each primer (Biomers, Ulm, Germany), 0.025 U/μl of HotMaster Taq (5Prime, Gaithersburg, MD, USA), 0.5 mg/μl molecular grade BSA (NEB, MA, USA), 12.5 ng DNA, filled up with HPLC H_2_O to a total volume of 250 μl. Each 250 μL PCR reaction mix was divided into five wells before the PCR. PCRs were run in a Biometra TAdvanced Thermocycler using the following program 94°C for 3 min, 40 cycles of 94°C for 30 s, 50°C for 30 s, and 65°C for 2 min, and 65°C for 5 min. The large reaction volume and BSA were necessary due to PCR inhibitors present in the samples. PCR products were purified and size selected (left sided) using SPRIselect with a ratio of 0.8× (Beckman Coulter, CA, USA) and quantified with a Qubit fluorometer (HS Kit, Thermo Fisher Scientific). Samples were pooled to equal molarity, and the final library purified with the MinElute Reaction Cleanup Kit (Qiagen, NL), as a precaution because the BSA used in the PCR caused adhesion of beads to the tube walls in the PCR clean‐up with SPRIselect. Paired‐end sequencing was performed on one lane of an Illumina HiSeq 2500 system with a rapid run 250‐bp PE v2 sequencing kit and 5% PhiX spike‐in. However, sequences contained ambiguous bases at two positions, due to air bubbles in the flow cell (SRR3399055). Thus, the run was repeated, this time loading two lanes with the same library with slightly different cluster densities, again with a 5% PhiX spike‐in.

We used the UPARSE pipeline in combination with custom R scripts (Dryad https://doi.org/10.5061/dryad.8v528) for data processing (Edgar, 2013; Fig. S5). Reads from both lanes were demultiplexed with a R script and paired end reads merged using Usearch v8.1.1861 –fastq_mergepairs with –fastq_maxdiffs and –fastq_maxdiffpct 99 (Edgar & Flyvbjerg, 2015). Primers were removed with Cutadapt version 1.9 on default settings (Martin, 2011). Sequences were trimmed to the same 217‐bp region amplified by the BF1 + BR1 primer set and the reverse complement build if necessary using fastx_truncate/fastx_revcomp. Only sequences with 207–227 bp were length used in further analysis (filtered with Cutadapt). Low quality sequences were then filtered from all samples using fastq_filter with maxee = 1. Sequences from all samples were then pooled, dereplicated (minuniquesize = 3) and clustered into operational taxonomic units (OTUs) using cluster_otus with 97% identity (Edgar, 2013) (includes chimera removal). A threshold of 97% was used to reduce the effect of sequencing errors, which might lead to the generation of additional “false” OTUs.

Preprocessed reads (Fig. S5, step B) of all samples were dereplicated again using derep_fulllength, but singletons were included. Sequences of each sample were matched against the OTUs with a minimum match of 97% using usearch_global. As the same library was loaded on both lanes, hit tables from both HiSeq lanes were combined, because they only represent sequencing replicates. Only OTUs with a read abundance above 0.01% in at least one sample were considered in downstream analysis. Within each sample, OTUs with less or equal than 0.01% were set to 0% sequence abundance to reduce the number of false positive OTUs. Taxonomy was assigned to the remaining OTUs using an R script searching the BOLD and NCBI database independently. Conflicting taxonomy was resolved on a case‐by‐case basis (with falling back to a coarser taxonomic level if the correct assignment was no evident). Only OTUs reliably identified as freshwater macroinvertebrates were included in the main analysis.

## 3 RESULTS

Weight measurements of the tissue used for DNA extraction were performed twice independently, with consistent results between replicates (*SD* = 0.083 mg). The library was sequenced on a HiSeq rapid run with a cluster density of 438 k/mm^2^ and 542 k/mm^2^ for lane 1 and 2 (raw data available on the NCBI SRA archive: SRR3399056 and SRR3399057). On average 1.71 (*SD* = 0.29, lane 1) and 2.17 (*SD* = 0.38, lane 2) million read pairs were obtained for each sample after demultiplexing (Fig. S4). Read quality varied with amplicon length and cluster density (Fig. S4) but did not affect results strongly as OTU abundance was very similar between both lanes (=sequencing replicates of identical library). However, stochastic effects between both lanes increased for OTUs with low read abundance (Fig. S6, variability between replicates for abundant OTUs > 10%, *SD* = 0.007, OTUs with 0.1–0.01 > % abundance, *SD* = 0.077).

The OTU raw data are available in Table S2 and morphology based identifications and taxa abundances in Table S3. After clustering and discarding low abundance OTUs, a total of 314 OTUs remained in the dataset (Fig. S7, Table S2). Approximately 71% of these OTUs could be reliably identified with available reference databases, with 58% of the OTUs belonging to freshwater macroinvertebrate taxa (Fig. S8). All high abundance OTUs (at least 0.1% of reads) were identified as macroinvertebrate. Of these taxa 45 of 52 were reliably identified at species level, of which about 3/4 had 100% similarity matches to reference sequences. Low abundance OTUs (<0.1%) often showed poor matches to databases or could not be identified at all (see Fig. S8). With DNA metabarcoding over twice as many macroinvertebrate taxa and in particular five times more species were detected than with morphology based identification (Fig. S9). The main stream (P8) and tributary (P10) could be clearly distinguished, with only 14.3% of OTUs shared between both sites (Fig. S7, 36.4% similarity based on morphological identification, Table S1).

**Figure 3:**
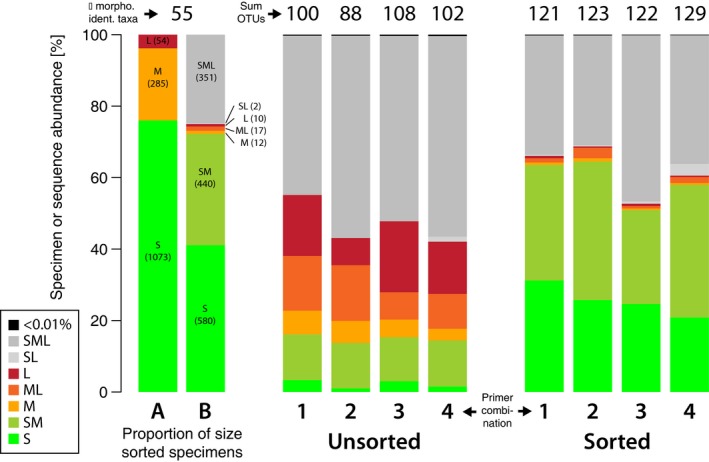
Comparison of specimen number in each size category against the respective OTU read abundance of unsorted and sorted samples with four different primer sets. The proportion of sorted specimens is shown in barplot A, while plot B is showing how many morphologically identified taxa are sharing the same size categories. For example, if a taxon is represented by small, medium, and large specimens it gets assigned to “SML” (grey) in the metabarcoding dataset, as specimens of all size classes contribute DNA which clusters into the same OTU regardless of specimen size. Thus, reads cannot always be reassigned to small, medium, or large specimens, but a combination of those (see also Fig. S7). Numbers above the plot give the total number of taxa identified with morphology and the number of OTUs detected with each primer set for unsorted and sorted samples. The numbers 1–4 below the plots indicate the different primer combinations used; 1 = BF1 + BR1, 2 = BF1 + BR2, 3 = BF2 + BR1, 4 = BF2 + BR2

**Figure 4:**
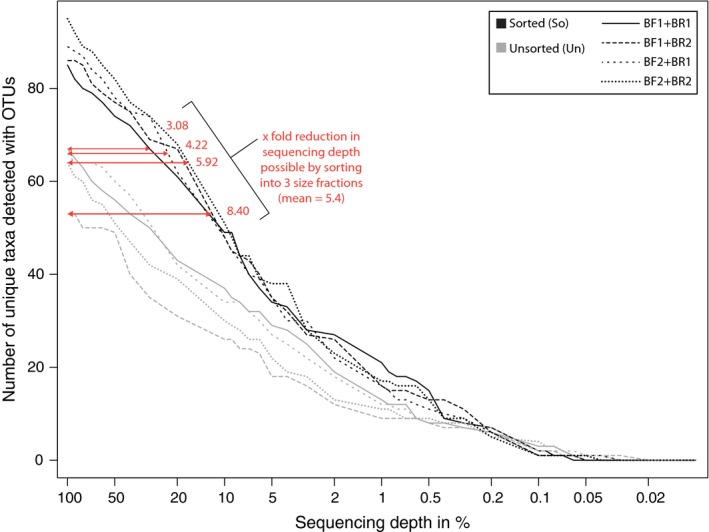
Amount of detected taxa based on OTUs with unsorted (Un) and sorted samples (So) for the four tested primer combinations, considering different sequencing depths. The sequencing depth is plotted on a logarithmic scale

Sorting the sample into three size categories and proportional pooling of DNA extracts by amount of specimens in each category reduced the dominance of large specimens substantially (Fig. 3). The sorted samples (So) resembled the composition of the original sample much better (average difference to original composition = 2.3‐fold, *SD* = 2.49) than the unsorted samples (Un, average difference = 9.0‐fold, *SD* = 7.88, Fig. 3). Using the four primer sets an average of 88.75 (*SD* = 6.46) invertebrate taxa were detected in the sorted samples, compared against 62.5 (*SD* = 4.5) in the unsorted samples (30% less, paired *t*‐test, *p* = .005, Fig. 4, no rarefaction applied). Using the S, M, and L samples as controls, we could estimate the expected (*E*) amount of taxa, we should be detecting with each primer pair (Fig. S10). In sorted samples (So), very similar amounts of taxa as in the controls (*E*) were detected (paired *t* test, *p* = .17). However, on average only 80% (*SD* = 8%) of the expected number of taxa were detected when the complete sample was extracted without sorting (Fig. S10a, paired *t* test, *p* < .001). The same trend was observed when looking at Shannon Diversity (Fig. S10b, paired *t* test, *E* vs. So; *p* = .9153, *E* vs. Un; *p* < .001). When comparing the taxa detected with metabarcoding against the taxa list based on morphological identification, again the unsorted samples showed decreased detection rates (67%, *SD* = 3%, paired *t* test, *p* = .006). However, also with sorting, only 74% (*SD* = 3%) of the morphologically identified taxa were detected with each primer set, which, however, was not significantly different than the detection rates in the controls *E* (paired *t* test, *p* = .23, Fig. S10c). Six morphologically identified taxa were not detected in our metabarcoding dataset (Fig. S7, Table S3, the morphologically determined “Plecoptera” and “Insecta” specimens are counted as “detected” here, as several insect and Plecoptera OTUs were present in the dataset. We, however, do not know which of the OTUs matches the morphological identified Plecoptera or Insecta). The reduced number of taxa detected with the unsorted samples persists when the sequencing depth is reduced (Fig. 4). Sample sorting does reduce the required sequencing depth to detect the same amount of taxa by ~5 times, compared to the unsorted samples.

## 4 DISCUSSION

### 4.1 Effects of sorting metabarcoding samples by specimen size

We sorted two samples by specimen size (resembling biomass) into small, medium, and large specimens and pooled them proportionately by specimen abundance per size class and compared the results against unsorted samples. Our results demonstrate that read abundances of the unsorted samples were dominated by few biomass rich taxa that contribute the majority of DNA in the bulk extraction. This does not only skew the read abundances in favor of biomass rich specimens, but also some smaller and less abundant taxa remained undetected (on average ~30% fewer taxa detected in the unsorted samples). The size‐sorted samples only need 1/5 of the sequencing depth to detect the same amount of taxa as in the unsorted samples. This means that sorting metabarcoding bulk samples by specimen size can substantially reduce sequencing costs, if the aim is to detect all taxa present in the sample regardless of biomass. While we only manually sorted our samples into three size categories, further cost reductions might be possible by sorting samples into more size categories. It is likely that larger specimens will have similar effects on metagenomic sequencing, and thus, sorting by specimen size and correcting for abundance might also likely be viable for these bulk samples.

Based on basic physical laws, it is expected that large specimens are overrepresented in a metabarcoding study when extracted in bulk together with smaller organisms. Thus, it is no real surprise that sorting by specimen size in combination with pooling fractions proportionally by number of specimens in each size category leads to a more equal representation of specimens in the sample and increased the detection of rare and small specimens with DNA metabarcoding. However, also the limitations and shortcomings of this study should be discussed here. While we took great care to reduce factors biasing ours result, for example by extracting all samples from the same digested tissue aliquots, we failed to adjust for the amount of tissue digested in these aliquots for the sorted mock samples (Fig. 2h,i). This leads to a slight underrepresentation of small taxa in the mock samples, as for medium and large taxa, more tissue was extracted (Fig. 2e). While this will not change the overall effects found in this study, it does mean that the positive effects of sample sorting are potentially slightly underestimated here: We hypothesize that with the correct (higher amount) of small specimens used in the mock communities, even more taxa could have been detected in the sorted samples. Additionally, this study was only carried out on two sampling sites and with limited morphological identifications. With more time spent and higher taxonomic expertise, probably more taxa could have been identified morphologically. Also, despite the COI reference databases being fairly complete for common macroinvertebrate taxa (Fig. S8), there are still gaps and potentially unreliable reference barcodes present, preventing the identification of some less abundant OTUs. We also show that with our dataset that stochastic effects during Illumina sequencing affect mainly low abundant OTUs, which was recently also confirmed in other studies (Leray & Knowlton, 2017). For a full and more detailed discussion of effects limitations of DNA metabarcoding for routine macroinvertebrate monitoring see Elbrecht et al. (2017). Nevertheless, DNA‐based identifications can be more accurate than classical morphology based identification (Stein et al., 2013; Sweeney et al., 2011) as we also show with our two kick samples in this project.

### 4.2 Implications: not all samples have to be sorted

While we could demonstrate and also quantify the increased resolution and potential cost savings by size sorting metabarcoding bulk samples, we have to acknowledge that these sample sorting steps can be time consuming and potentially also a source of cross‐contamination between samples. Thus, we do not recommend sorting every sample by specimen biomass right away. First of all, the sample should have specimens varying several magnitudes in biomass. If all specimens have similar sizes, sorting will likely not improve metabarcoding results. Additionally, the number of samples which can be reliably tagged on a HTS run in combination with the expected sequencing output, might make sorting obsolete if expected sequencing depth per sample is sufficiently high. However, in many cases where bulk samples show substantial variation in biomass, sequencing depth should be sufficiently high to also detect small and rare taxa. Here, sorting samples and adjusting for specimen biomass can help to increase the number of taxa detected, making it possible to pool more samples on the same sequencing run.

Whether or not the method of size sorting should be used in a study depends on sample composition and characteristics as discussed above, but more importantly it should be considered if it is necessary to detect small and rare taxa present in the bulk sample (e.g., for non‐targeted early detection of pests, invasive species or to build barcoding reference databases). It has to be stressed that for most studies, the proportion of the abundant taxa is most relevant, which gets distorted by sample sorting and pooling by abundance of small, medium, and large specimens. Also, if samples just contain a few large specimens and abundance data are not that important, one could retain a small piece of tissue in the sample (e.g., a leg of an invertebrate) and remove the rest of the specimen from the sample (as performed e.g. by Ji et al., 2013). Especially, if only presence–absence data are desired, this might be a good trade‐off to reduce the negative influence of a few large specimens on the dataset, without sorting the complete sample. However, treating samples to reduce the influence of biomass rich specimens should be performed systematically across samples to not introduce processing biases. In this study, sorted individual specimens into three size categories under a stereo microscope to get very accurate size classes needed to test this method. With approximately 2–3 hrs for each sample and the additional workload for DNA extraction, this is a highly time consuming step, making the technique of size sorting samples impractical for large sample quantities. Studies on marine invertebrate did size sort samples by sieving the samples with different sieve sizes from 63 μm to 10 mm (Leray & Knowlton, 2015; Wangensteen & Turon, 2016). Sieving is probably the only feasible method for processing large numbers of samples, but care has to be taken when cleaning the sieves between samples, to prevent cross‐contamination. Sieving might also change the community composition as very small bacteria on surfaces and small organism might get lost, and broken off body parts (e.g., legs, antennae) or tissue parts from prey animals might end up in the lowest size fraction (Leray & Knowlton, 2015; Wangensteen & Turon, 2016). These effects have to be taken into consideration when looking at each size fraction individually. However, if the goal is to obtain a presence–absence taxa list for a complete sample, sieving and proportional pooling might be an ideal solution to minimize bias introduced by large specimens in the samples. Using dry specimen weight from each size fraction can be used to roughly estimate the number of taxa in each size fraction, which can then be used to pool the DNA proportionately, instead of sequencing each size fraction individually. Also, precise pooling of digested tissue (Fig. 1c) as performed in this ground truthing study might not be needed for routine application of this size sorting approach. Depending on the accuracy needed, pooling ground tissue from different size fractions directly (Fig. 1b) or extracting, quantifying, and pooling of DNA (Fig. 1d) might already be sufficient to reduce the prevalence of large specimens in the dataset.

## 5 CONCLUSIONS

We demonstrated that sorting metabarcoding samples into three specimen size categories and then pooling the tissue fractions proportionally to the number of specimens in each size class can reduce the amount of required sequencing depth compared to the unsorted sample by 80%. Sample sorting leads to a more balanced taxa assessment, dramatically reducing the overrepresentation of large specimens on the dataset. While size sorting of bulk samples might not be necessary or suitable for all samples, ecosystems or research questions, we encourage to evaluate if sample fractioning could be beneficial and feasible in respective metabarcoding projects. Also, some metagenomic projects will likely profit from presorting samples by biomass, even though we did not explicitly test this here.

## CONFLICT OF INTEREST

The authors report no conflicts of interest. The authors alone are responsible for the content and writing of the manuscript.

## AUTHOR CONTRIBUTIONS

VE, BP and FL conceived the ideas and designed methodology; BP collected and identified specimens and carried out the laboratory work; VE performed bioinformatic analyzes and wrote the manuscript. All authors contributed critically to the drafts and gave final approval for publication.
