# Supplementary material for: Sorting things out: Assessing effects of unequal specimen biomass on DNA metabarcoding
Source: Ecol Evol. 2017 Jul 28;7(17):6918–26. doi: 10.1002/ece3.3192 (PMC5587478; doi:10.1002/ece3.3192)
Supplement: Supplementary file 1 [file ECE3-7-6918-s001.pdf]

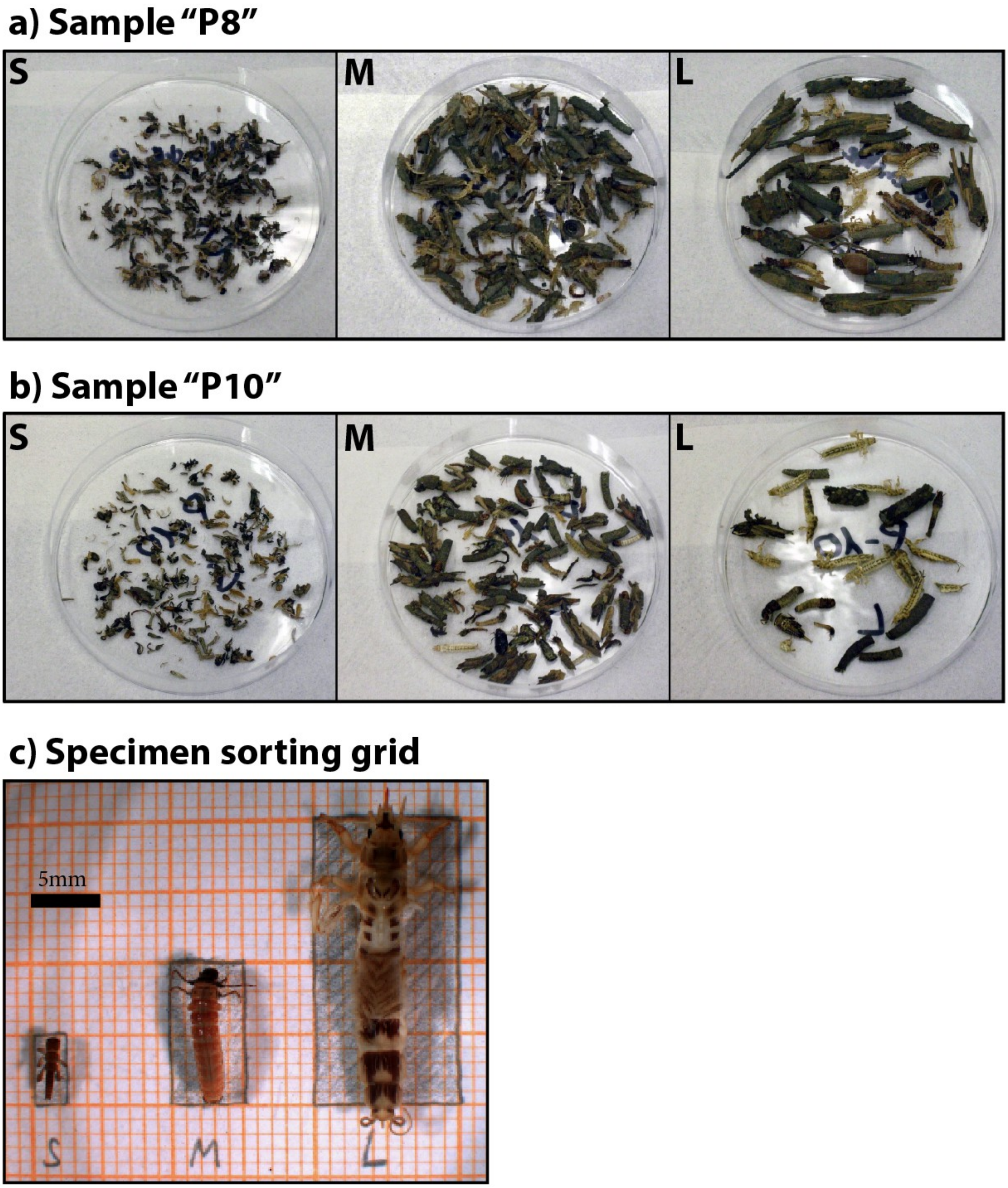

Figure S1: Overview of specimens from both sample sites (P8: **a**, P10: **b**) sorted by biomass into small (S), medium (M) and large (L) size categories using a sorting grid (**c**).
