# Supplementary material for: Sorting things out: Assessing effects of unequal specimen biomass on DNA metabarcoding
Source: Ecol Evol. 2017 Jul 28;7(17):6918–26. doi: 10.1002/ece3.3192 (PMC5587478; doi:10.1002/ece3.3192)
Supplement: Supplementary file 2 [file ECE3-7-6918-s002.pdf]

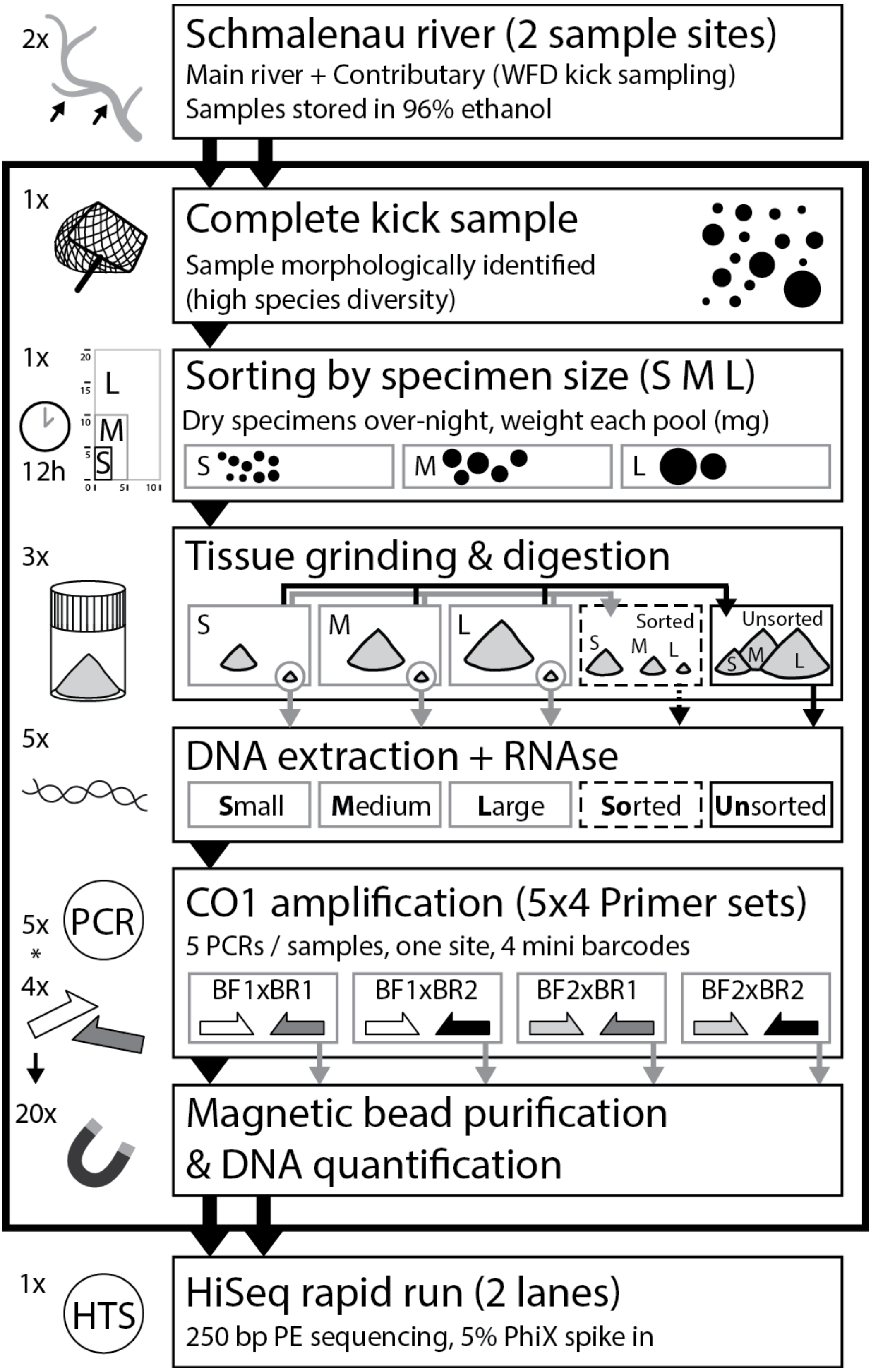

Figure S2: Overview of the sample processing and laboratory steps carried out in this metabarcoding study. Specimens from 2 sample locations were sorted by specimen size into 3 size classes (S, M, L). The tissue of each class was then digested and pooled based on number of taxa in each class or total specimen weight, to simulate sorted (So) and unsorted samples (Un). Each sample was then amplified with the same 4 primer combinations using a fusion primer PCR protocol, and the library sequenced on 2 HiSeq rapid run lanes.
