# Supplementary material for: Sorting things out: Assessing effects of unequal specimen biomass on DNA metabarcoding
Source: Ecol Evol. 2017 Jul 28;7(17):6918–26. doi: 10.1002/ece3.3192 (PMC5587478; doi:10.1002/ece3.3192)
Supplement: Supplementary file 3 [file ECE3-7-6918-s003.pdf]

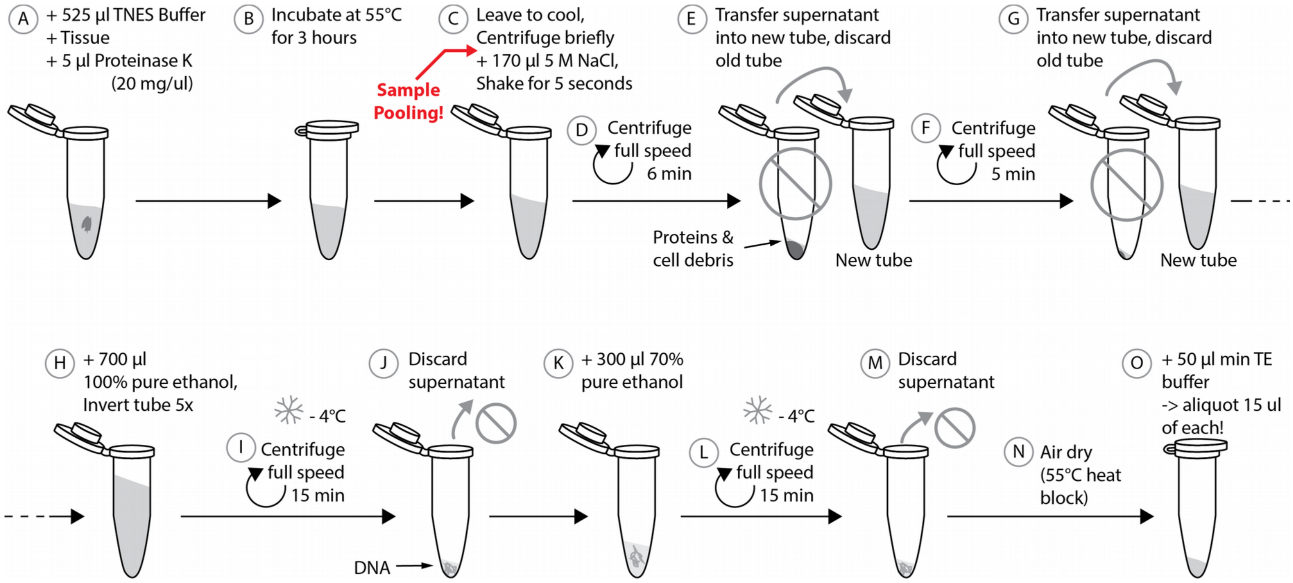

Figure S3: Modified salting out DNA extraction protocol (based on Sunnucks & Hales 1996). In step C digestion solution from S, M and L were used in the right proportion to create the sorted (So) and unsorted (Un) samples.

Reference: Sunnucks & Hales (1996). Numerous transposed sequences of mitochondrial cytochrome oxidase I-II in aphids of the genus *Sitobion* (Hemiptera: Aphididae). Molecular biology and evolution 13:510–524.

TNES buffer (**T**ris, **N**aCl, **E**DTA, **S**DS):
50 mM Tris, pH 7.5
400 mM NaCl
20 mM EDTA
0.5 % SDS

min TE buffer (pH 8)
10 mM Tris Base
0.1 mM EDTA
