# Supplementary material for: Sorting things out: Assessing effects of unequal specimen biomass on DNA metabarcoding
Source: Ecol Evol. 2017 Jul 28;7(17):6918–26. doi: 10.1002/ece3.3192 (PMC5587478; doi:10.1002/ece3.3192)
Supplement: Supplementary file 4 [file ECE3-7-6918-s004.pdf]

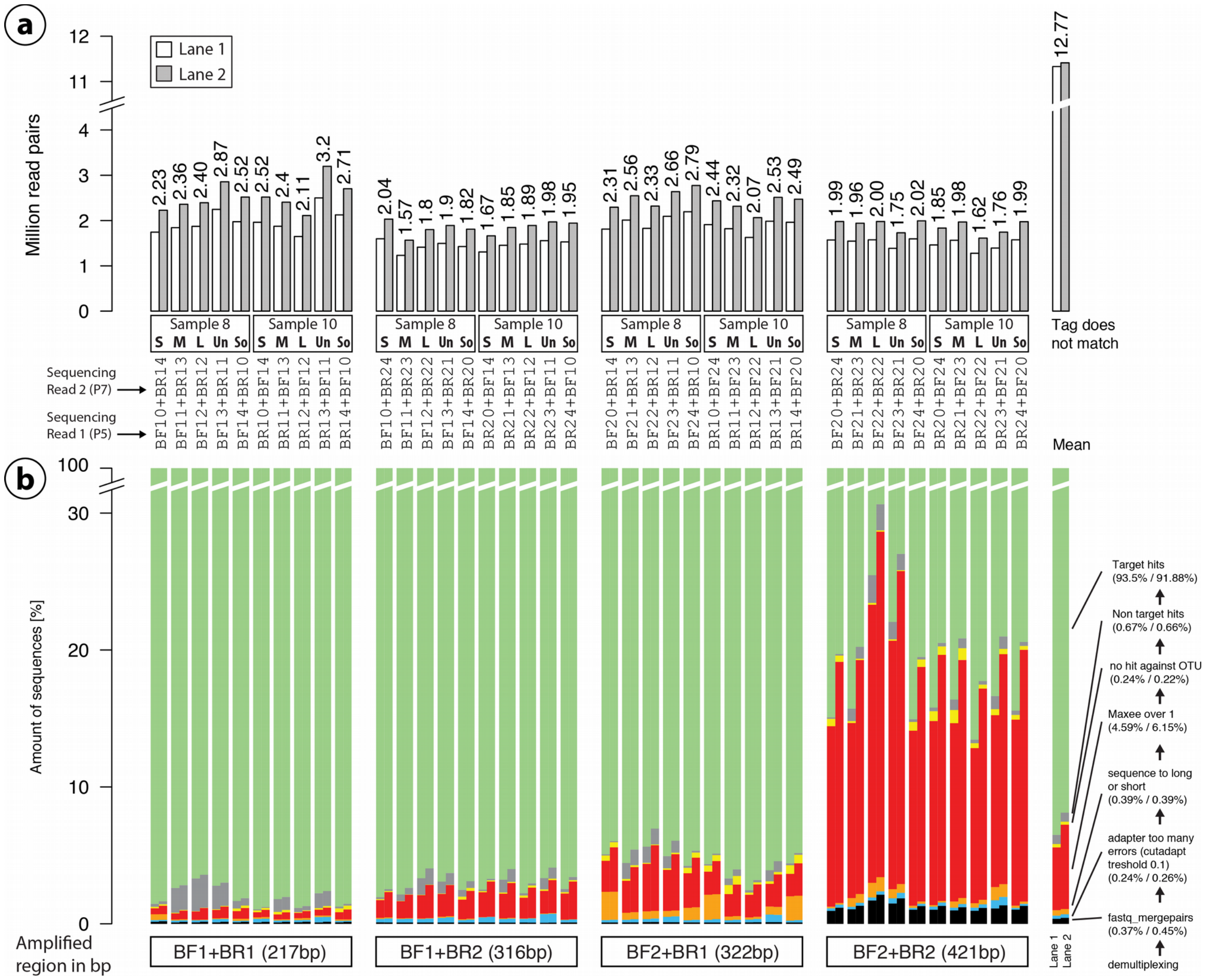

Figure S4: Overview of used primer (tagging combinations) with obtained read pairs per sample and amount of sequences discarded by bioinformatic processing. **a)** Number of paired end reads obtained for each sample in each sequencing lane. Numbers above bars give the proportion of reads in percentage. **b)** Amount of sequences excluded in each major bioinformatic processing step for each sample. Size of the amplified region (not the fragment size) is given below in boxes for each primer combination.
