# Supplementary material for: Sorting things out: Assessing effects of unequal specimen biomass on DNA metabarcoding
Source: Ecol Evol. 2017 Jul 28;7(17):6918–26. doi: 10.1002/ece3.3192 (PMC5587478; doi:10.1002/ece3.3192)
Supplement: Supplementary file 5 [file ECE3-7-6918-s005.pdf]

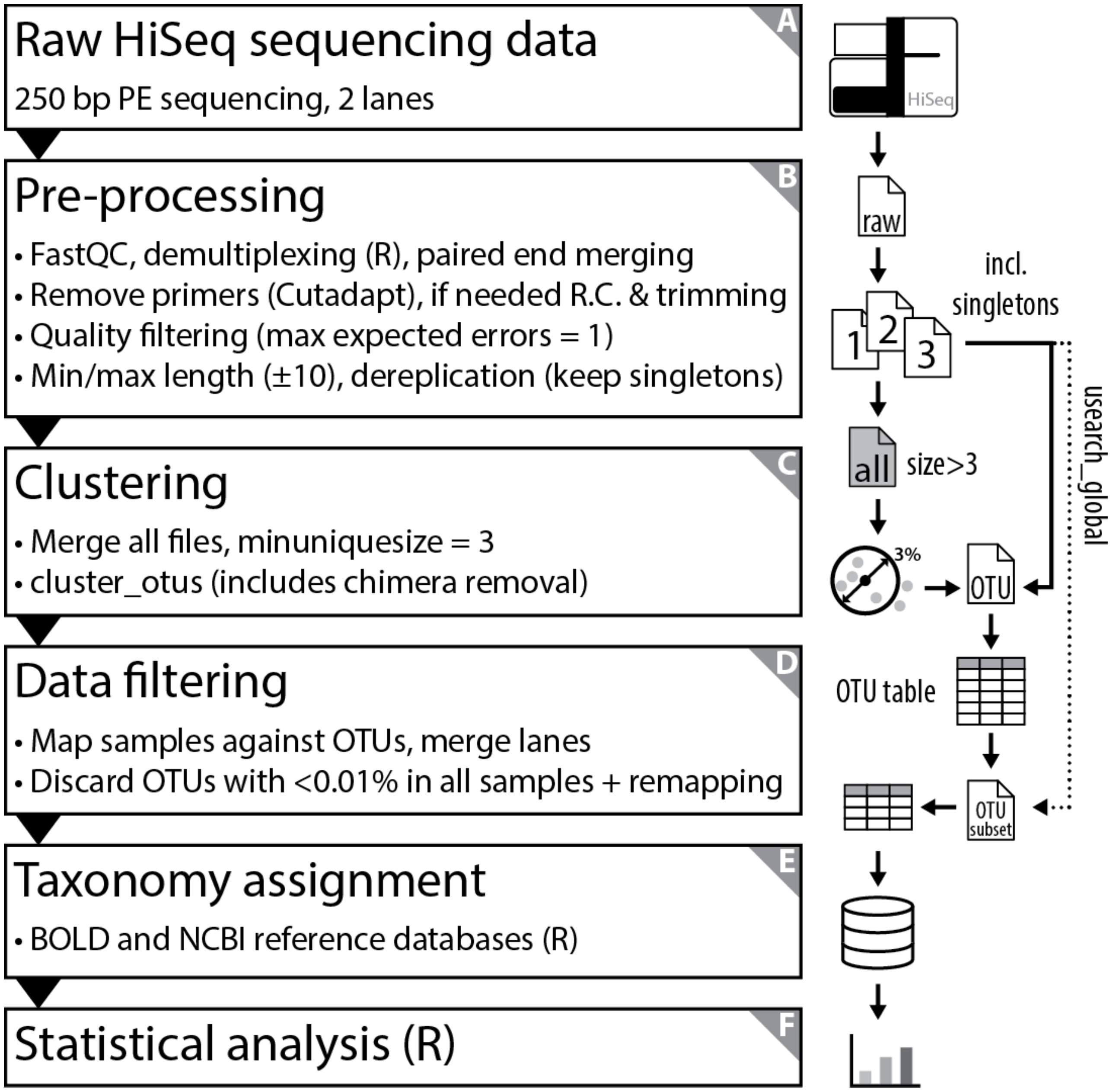


**Figure S5**: Detailed overview of the bioinformatic processing of the Illumina hight throughput sequencing data. Raw sequence data (**A**) is demultiplexed and pre-processed (paired end merging, remove primers, trimming, reverse complement, removal of low quality reads) (**B**). The processed sequences are then pooled and demultiplexed with a minimum size of 3, to reduce noise by sequencing errors in clustering (**C**). Reads from all samples are then compared against the generated OTUs and OTUs with a minimum of 0.01% of sequences assigned in at least one sample are discarded (data from both lanes is merged at this point) (**D**). All reads are again mapped against the OTU subset to generate the final OTU table, with taxonomy being assigned to each centroid using NCBI and BOLD (**E**).
