# Supplementary material for: Sorting things out: Assessing effects of unequal specimen biomass on DNA metabarcoding
Source: Ecol Evol. 2017 Jul 28;7(17):6918–26. doi: 10.1002/ece3.3192 (PMC5587478; doi:10.1002/ece3.3192)
Supplement: Supplementary file 6 [file ECE3-7-6918-s006.pdf]

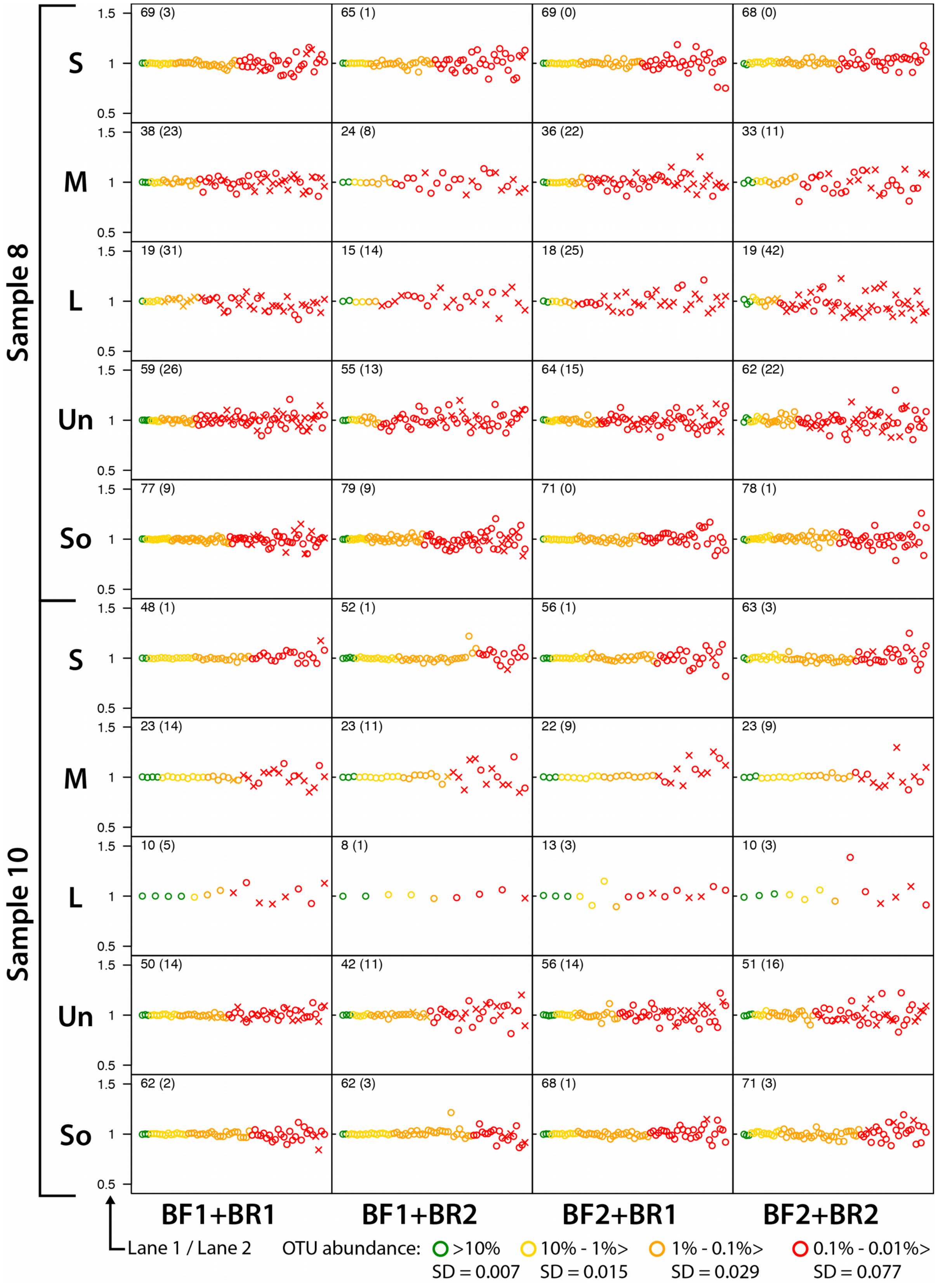

Figure S6: Number of sequences per OTU from sequencing lane 1 divided by lane 2 (minimum sequence abundance per OTU, from both replicates combined 0.01%). OTU abundance is indicated by colour, with the total number of target OTUs per sample and primer combination is given on the top left corner in each plot (number of non macroinvertebrate OTUs in brackets). Macroinvertebrate hits are indicated with circles (“o”), while other hits and unidentified OTUs are indicated by crosses in the plot (“x”). Standard deviation is given using all data points of the in the 4 OTU abundance classes.
