# Supplementary material for: Sorting things out: Assessing effects of unequal specimen biomass on DNA metabarcoding
Source: Ecol Evol. 2017 Jul 28;7(17):6918–26. doi: 10.1002/ece3.3192 (PMC5587478; doi:10.1002/ece3.3192)
Supplement: Supplementary file 7 [file ECE3-7-6918-s007.pdf]

OTU	Order (or coarser)	Family	Genus	Species	Sample 8 S	Sample 8 M	Sample 8 L	Sample 10 S	Sample 10 M	Sample 10 L
OTU_120	Trombidiformes	Lebertiidae								
OTU_123	Trombidiformes	Anystidae								
OTU_178	Trichoptera	Sericostomatidae	Oecismus	monedula						
OTU_111	Trichoptera	Hydropsychidae								
OTU_19	Trichoptera	Philopotamidae	Wormaldia	occipitalis	0	0	0	5	5	0
OTU_14	Trichoptera	Sericostomatidae	Sericostoma	personatum	20	2	0	19	3	0
OTU_2580	Trichoptera	Sericostomatidae	Sericostoma	personatum						
OTU_21	Trichoptera	Sericostomatidae	Sericostoma	baeticum						
OTU_153	Trichoptera	Sericostomatidae	Sericostoma							
OTU_1432	Trichoptera	Limnephilidae	Potamophylax	cingulatus	0	0	0	0	0	2
OTU_16	Trichoptera	Limnephilidae	Potamophylax	cingulatus						
OTU_1773	Trichoptera	Limnephilidae	Potamophylax	cingulatus						
	Trichoptera	Polycentropodidae	Polycentropus	kingi	2	5	0	0	0	0
OTU_48	Trichoptera	Polycentropodidae	Polycentropus	irroratus	1	2	0	0	0	0
OTU_38	Trichoptera	Polycentropodidae	Polycentropus	flavomaculatus	0	1	0	0	0	0
OTU_412	Trichoptera	Polycentropodidae	Polycentropus	flavomaculatus						
	Trichoptera	Philopotamidae			0	0	0	7	0	0
OTU_4	Trichoptera	Odontoceridae	Odontocerum	albicorne	0	1	1	2	12	0
OTU_157	Trichoptera	Limnephilidae	Halesus	radiatus	0	0	8	0	0	0
OTU_3353	Trichoptera	Limnephilidae	Halesus	radiatus						
OTU_6	Trichoptera	Limnephilidae	Halesus	radiatus						
OTU_20	Trichoptera	Limnephilidae	Drusus	annulatus	0	0	0	0	1	0
OTU_32	Trichoptera	Polycentropodidae	Cyrnus	trimaculatus	0	2	0	0	0	0
OTU_4149	Trichoptera	Limnephilidae	Chaetopteryx	villosa	1	36	1	8	29	1
OTU_2	Trichoptera	Limnephilidae	Chaetopteryx	villosa						
OTU_6059	Trichoptera	Limnephilidae	Chaetopteryx							
OTU_99	Trichoptera	Limnephilidae	Anomalopterygella	chauviniana	0	1	0	0	0	0
OTU_217	Symphypleona	Dicyrtomidae	Dicyrtomina	minuta						
OTU_205	Rhynchobdellida	Glossiphoniidae	Helobdella	stagnalis	0	1	0	0	0	0
OTU_212	Ploima									
OTU_291	Plecoptera									
OTU_375	Plecoptera	Perlodidae								
OTU_49	Plecoptera	Perlodidae	Diura	nanseni						
OTU_1553	Plecoptera	Perlodidae	Diura							
OTU_1249	Plecoptera	Leuctridae								
OTU_1641	Plecoptera	Leuctridae								
OTU_43	Plecoptera	Chloroperlidae	Siphonoperla	torrentium	0	0	0	1	0	0
OTU_5	Plecoptera	Nemouridae	Protonemura	auberti	0	0	0	54	6	0
	Plecoptera				0	0	0	1	0	0
OTU_175	Plecoptera	Nemouridae	Nemoura	avicularis	1	0	0	0	0	0
OTU_13	Plecoptera	Leuctridae	Leuctra	braueri	0	0	0	38	3	0
OTU_27	Plecoptera	Leuctridae	Leuctra	fusca	61	0	0	8	0	0
OTU_88	Plecoptera	Leuctridae	Leuctra	digitata						
OTU_73	Plecoptera	Leuctridae	Leuctra	aurita						
OTU_41	Plecoptera	Leuctridae	Leuctra	albida						
OTU_67	Plecoptera	Leuctridae	Leuctra							
	Plecoptera	Perlodidae	Dictyogenus		0	0	0	19	0	0
OTU_220	Philodinida	Philodinidae								
	Oligochaeta				9	0	0	22	3	0
OTU_114	Odonata	Cordulegastridae	Cordulegaster	boltonii	1	0	0	0	0	0
OTU_152	Odonata	Calopterygidae	Calopteryx	maculata						
	Nematoda				1	0	1	0	0	0
OTU_1067	Lumbriculida									
OTU_130	Lumbriculida									
OTU_162	Lumbriculida	Lumbriculidae								
OTU_4070	Lumbriculida	Lumbriculidae	Stylodrilus	heringianus						
OTU_75	Lumbriculida	Lumbriculidae	Stylodrilus	heringianus						
OTU_28	Lumbriculida	Lumbriculidae	Stylodrilus	heringianus						
OTU_54	Lumbriculida	Lumbriculidae	Stylodrilus	heringianus						
OTU_17	Lumbriculida	Lumbriculidae	Lumbriculus	variegatus						
OTU_69	Lumbriculida	Lumbriculidae	Lumbriculus							
	Insecta				1	0	0	2	0	0
OTU_272	Hymenoptera									
OTU_160	Hymenoptera	Formicidae	Myrmica	ruginodis						
OTU_159	Hymenoptera	Formicidae	Formicidae							
OTU_23	Hygrophila	Lymnaeidae	Radix	labiata	0	1	3	0	0	0
OTU_93	Hygrophila	Planorbidae	Ancylus	fluviatilis	1	2	0	0	0	0
OTU_35	Hemiptera	Veliidae	Velia	caprai	0	0	0	1	0	0
OTU_4226	Hemiptera	Micronectidae	Micronecta	poweri	8	0	0	0	0	0
OTU_83	Hemiptera	Corixidae	Micronecta		0	0	0	1	0	0
OTU_183	Harpacticoida									
OTU_167	Haplotaxida									
OTU_84	Haplotaxida	Tubificidae								
OTU_72	Haplotaxida	Tubificidae	Nais	communis variabilis						
OTU_112	Haplotaxida	Tubificidae	Nais	alpina						
OTU_265	Haplotaxida	Lumbricidae								
OTU_15	Haplotaxida	Lumbricidae	Eiseniella	tetraedra						
OTU_97	Haplotaxida	Enchytraeidae								
OTU_233	Ephemeroptera	Leptophlebiidae	Leptophlebia	marginata	63	0	0	26	0	0
OTU_7	Ephemeroptera	Leptophlebiidae	Habrophlebia	lauta	10	0	0	4	0	0
OTU_194	Ephemeroptera	Leptophlebiidae	Habrophlebia	lauta						
OTU_1086	Ephemeroptera	Leptophlebiidae	Habrophlebia							
OTU_26	Ephemeroptera	Leptophlebiidae	Habroleptoides		0	0	0	14	0	0
OTU_2676	Ephemeroptera	Ephemerellidae			212	2	0	0	0	0
OTU_2130	Ephemeroptera	Ephemerellidae	Serratella							
OTU_630	Ephemeroptera	Ephemerellidae	Serratella							
OTU_30	Ephemeroptera	Ephemerellidae	Ephemerella	ignita						
OTU_3571	Ephemeroptera	Ephemerellidae	Ephemerella	ignita						
OTU_4831	Ephemeroptera	Ephemerellidae	Ephemerella	ignita						
OTU_8	Ephemeroptera	Ephemerellidae	Ephemerella	ignita						
OTU_102	Ephemeroptera	Ephemeridae			62	90	13	2	8	10
OTU_2966	Ephemeroptera	Ephemeridae	Ephemera	danica						
OTU_1	Ephemeroptera	Ephemeridae	Ephemera	danica						
OTU_255	Ephemeroptera	Ephemeridae	Ephemera							
OTU_2108	Ephemeroptera	Ephemeridae	Ephemera							
OTU_3	Ephemeroptera	Heptageniidae	Ecdyonurus	venosus	0	0	0	0	12	1
OTU_24	Ephemeroptera	Heptageniidae	Ecdyonurus							
OTU_79	Ephemeroptera	Baetidae	Centroptilum	luteolum	6	0	0	0	0	0
OTU_59	Ephemeroptera	Baetidae	Centroptilum	luteolum						
OTU_113	Ephemeroptera	Baetidae	Baetis	scambus	13	0	0	38	0	0
OTU_11	Ephemeroptera	Baetidae	Baetis	rhodani vernus						
OTU_22	Ephemeroptera	Baetidae	Baetis	rhodani						
OTU_85	Ephemeroptera	Baetidae	Baetis	rhodani						
OTU_57	Ephemeroptera	Baetidae	Baetis	rhodani						
OTU_294	Ephemeroptera	Baetidae	Baetis	rhodani						
OTU_12	Ephemeroptera	Baetidae	Baetis	muticus						
OTU_356	Ephemeroptera	Baetidae	Baetis	muticus						
OTU_253	Diptera									
OTU_144	Diptera									
OTU_142	Diptera									
OTU_90	Diptera									
OTU_125	Diptera									
OTU_456	Diptera	Sciaridae								
OTU_53	Diptera	Scathophagidae								
OTU_29	Diptera	Thaumaleidae	Thaumalea	testacea	0	0	0	9	0	0
OTU_44	Diptera	Tachinidae			7	5	0	0	1	0
OTU_2219	Diptera	Tabanidae								
OTU_237	Diptera	Tabanidae								
OTU_210	Diptera	Tabanidae								
OTU_191	Diptera	Tabanidae								
OTU_185	Diptera	Tabanidae								
OTU_155	Diptera	Tabanidae								
OTU_789	Diptera	Tabanidae								
OTU_124	Diptera	Tabanidae								
OTU_154	Diptera	Tabanidae								
OTU_6076	Diptera	Tabanidae								
OTU_141	Diptera	Tabanidae								
OTU_9	Diptera	Tabanidae	Tabanus	cordiger						
OTU_259	Diptera	Tabanidae	Hybomitra	epistates						
OTU_33	Diptera	Tabanidae	Hybomitra	distinguenda						
OTU_2217	Diptera	Simuliidae	Simulium	vernum	5	0	0	65	0	0
OTU_64	Diptera	Simuliidae	Simulium	vernum						
OTU_77	Diptera	Simuliidae	Simulium	velutinum aureum						
OTU_50	Diptera	Simuliidae	Simulium	urbanum brevidens cryophilum						
OTU_86	Diptera	Simuliidae	Simulium	ornatum						
OTU_10	Diptera	Simuliidae	Simulium	cryophilum vernum						
OTU_39	Diptera	Pediciidae	Pedicia	littoralis	0	1	0	0	4	0
OTU_92	Diptera	Limoniidae	Eloeophila	trimaculata	0	0	0	1	2	0
OTU_52	Diptera	Limoniidae	Eloeophila							
OTU_74	Diptera	Dixidae	Dixa		0	0	0	13	0	0
OTU_61	Diptera	Pediciidae	Dicranota	pavida	0	0	0	1	7	0
OTU_46	Diptera	Pediciidae	Dicranota	lucidipennis						
OTU_94	Diptera	Chironomidae			43	0	0	80	0	0
OTU_70	Diptera	Chironomidae								
OTU_170	Diptera	Chironomidae								
OTU_103	Diptera	Chironomidae								
OTU_42	Diptera	Chironomidae								
OTU_91	Diptera	Chironomidae								
OTU_87	Diptera	Chironomidae	Zavrelimyia							
OTU_133	Diptera	Chironomidae	Tvetenia	calvescens						
OTU_34	Diptera	Chironomidae	Trissopelopia	longimana						
OTU_51	Diptera	Chironomidae	Trissopelopia	flavida						
OTU_201	Diptera	Chironomidae	Rheocricotopus	fuscipes						
OTU_104	Diptera	Chironomidae	Prodiamesa	olivacea						
OTU_2981	Diptera	Chironomidae	Prodiamesa							
OTU_148	Diptera	Chironomidae	Potthastia	longimanus						
OTU_107	Diptera	Chironomidae	Polypedilum	pullum						
OTU_135	Diptera	Chironomidae	Polypedilum	convictum						
OTU_78	Diptera	Chironomidae	Paratrissocladius	excerptus						
OTU_117	Diptera	Chironomidae	Orthocladius	dentifer						
OTU_228	Diptera	Chironomidae	Microtendipes	pedellus						
OTU_96	Diptera	Chironomidae	Micropsectra	pallidula						
OTU_63	Diptera	Chironomidae	Micropsectra	notescens						
OTU_58	Diptera	Chironomidae	Macropelopia	notata						
OTU_76	Diptera	Chironomidae	Heterotrissocladius	marcidus						
OTU_62	Diptera	Chironomidae	Epoicocladius	ephemerae						
OTU_129	Diptera	Chironomidae	Corynoneura							
OTU_81	Diptera	Chironomidae	Conchapelopia	hittmairorum						
OTU_219	Diptera	Chironomidae	Brillia	longifurca						
OTU_132	Diptera	Chironomidae	Brillia	bifida						
	Diptera	Ceratopogonidae			1	0	0	1	0	0
OTU_169	Diplura				0	0	0	2	0	0
OTU_31	Coleoptera	Elmidae	Limnius	volckmari	11	0	0	1	0	0
OTU_71	Coleoptera	Elmidae	Limnius	perrisi						
OTU_56	Coleoptera	Hydraenidae	Hydraena	gracilis	1	0	0	2	0	0
OTU_47	Coleoptera	Elmidae	Oulimnius	tuberculatus	6	0	0	0	0	0
OTU_60	Coleoptera	Elmidae	Elmis	maugetii						
OTU_55	Coleoptera	Elmidae	Elmis	aenea						
OTU_40	Coleoptera	Dytiscidae	Deronectes	latus angusi	5	0	0	1	0	0
OTU_582	Coleoptera				29	0	0	16	0	0
OTU_18	Coleoptera	Scirtidae	Hydrocyphon	deflexicollis						
OTU_101	Coleoptera	Scirtidae	Elodes	minuta						
OTU_82	Coleoptera	Scirtidae	Elodes	minuta						
OTU_25	Coleoptera	Scirtidae	Elodes	marginata						
OTU_66	Coleoptera	Nitidulidae	Brassicogethes	aeneus						
OTU_256	Coleoptera	Curculionidae	Orchestes	fagi						
OTU_45	Coleoptera	Hydrophilidae	Anacaena	globulus	2	0	0	1	0	0
OTU_229	Coleoptera	Dytiscidae	Agabus		0	0	0	0	1	0
OTU_36	Coleoptera	Dytiscidae	Agabus							
OTU_37	Chordodea	Parachordodidae								
Below_0.01										

OTUs below 0.01% abundance and non freshwater macroinvertebrate taxa →

Primer combinations: BF1+BR1 | BF1+BR2 | BF2+BR1 | BF2+BR2 (each for Sample 8 and Sample 10; columns S, M, L, Un, So)

BF10_BR14, BF11_BR13, BF12_BR12, BF13_BR11, BF14_BR10, BF10_BR24, BF11_BR22, BF12_BR22, BF13_BR21, BF14_BR20, BF20_BR14, BF21_BR13, BF22_BR12, BF23_BR11, BF24_BR10, BF20_BR24, BF21_BR23, BF22_BR22, BF23_BR21, BF24_BR20, BR10_BF14, BR11_BF13, BR12_BF12, BR13_BF11, BR14_BF10, BR20_BF14, BR21_BF13, BR22_BF12, BR23_BF11, BR24_BF10, BR10_BF24, BR11_BF23, BR12_BF22, BR13_BF21, BR14_BF20, BR20_BF24, BR21_BF23, BR22_BF22, BR23_BF21, BR24_BF20

Figure S7: OTU table showing relative abundance [%] of freshwater macroinvertebrate reads across the different samples and primer combinations. Taxonomy based on NCBI and BOLD reference databases is given on the left, with morphological identifications indicated with green. When taxa were not detected in the sequence data they have a red background, and one case where determination based on morphology did give a higher taxonomic resolution than based on sequence data is indicated Yellow. The amount of taxa sorted into small (S), medium (M) and large (L) is indicated in blue boxes for both sample sites. For each primer combination the sequence abundances for S, M, L as well as the complete **un**sorted sample (Un) and the **so**rted sample (So) is shown, with read abundance underlayed by color.

Legend
> 10 %
10-5
5-1
1-0.5
0.5-0.1
0.1-0.05
0.05-0.01
0.01-0.001
