# Supplementary material for: Sorting things out: Assessing effects of unequal specimen biomass on DNA metabarcoding
Source: Ecol Evol. 2017 Jul 28;7(17):6918–26. doi: 10.1002/ece3.3192 (PMC5587478; doi:10.1002/ece3.3192)
Supplement: Supplementary file 8 [file ECE3-7-6918-s008.pdf]

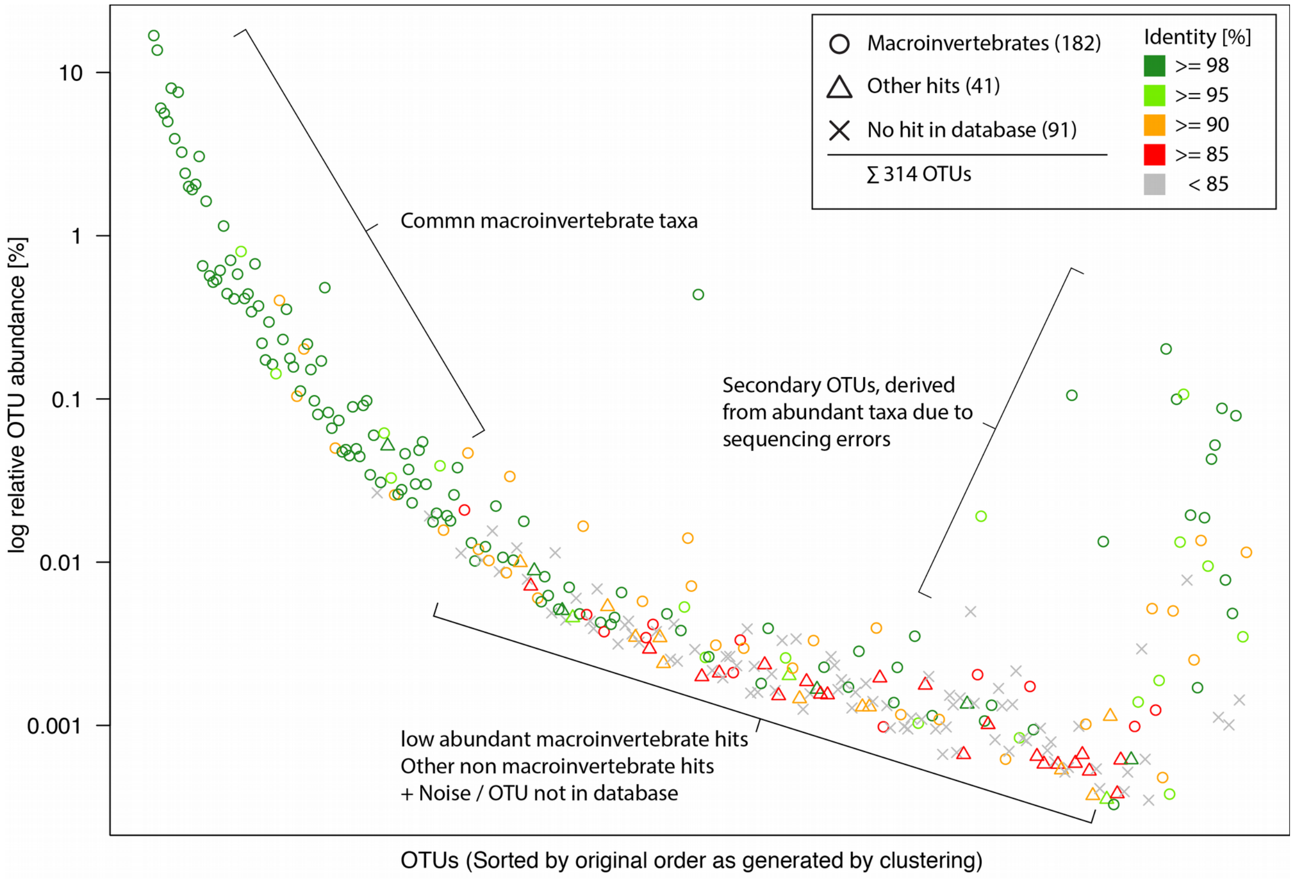

Figure S8: Relative log OTU abundance with indication of % match to the BOLD or NCBI reference databases. Order of OTUs as determined by the original clustering with Usearch. Target invertebrate taxa are represented by circles, other hits by triangles and OTUs without matching reference sequences with crosses. Some of the “ho hit in database” had matches to uncultured fungi (See table S2), which however is not considered as a valid hit and thus the % match was set to < 85%.
