# Supplementary material for: Sorting things out: Assessing effects of unequal specimen biomass on DNA metabarcoding
Source: Ecol Evol. 2017 Jul 28;7(17):6918–26. doi: 10.1002/ece3.3192 (PMC5587478; doi:10.1002/ece3.3192)
Supplement: Supplementary file 9 [file ECE3-7-6918-s009.pdf]

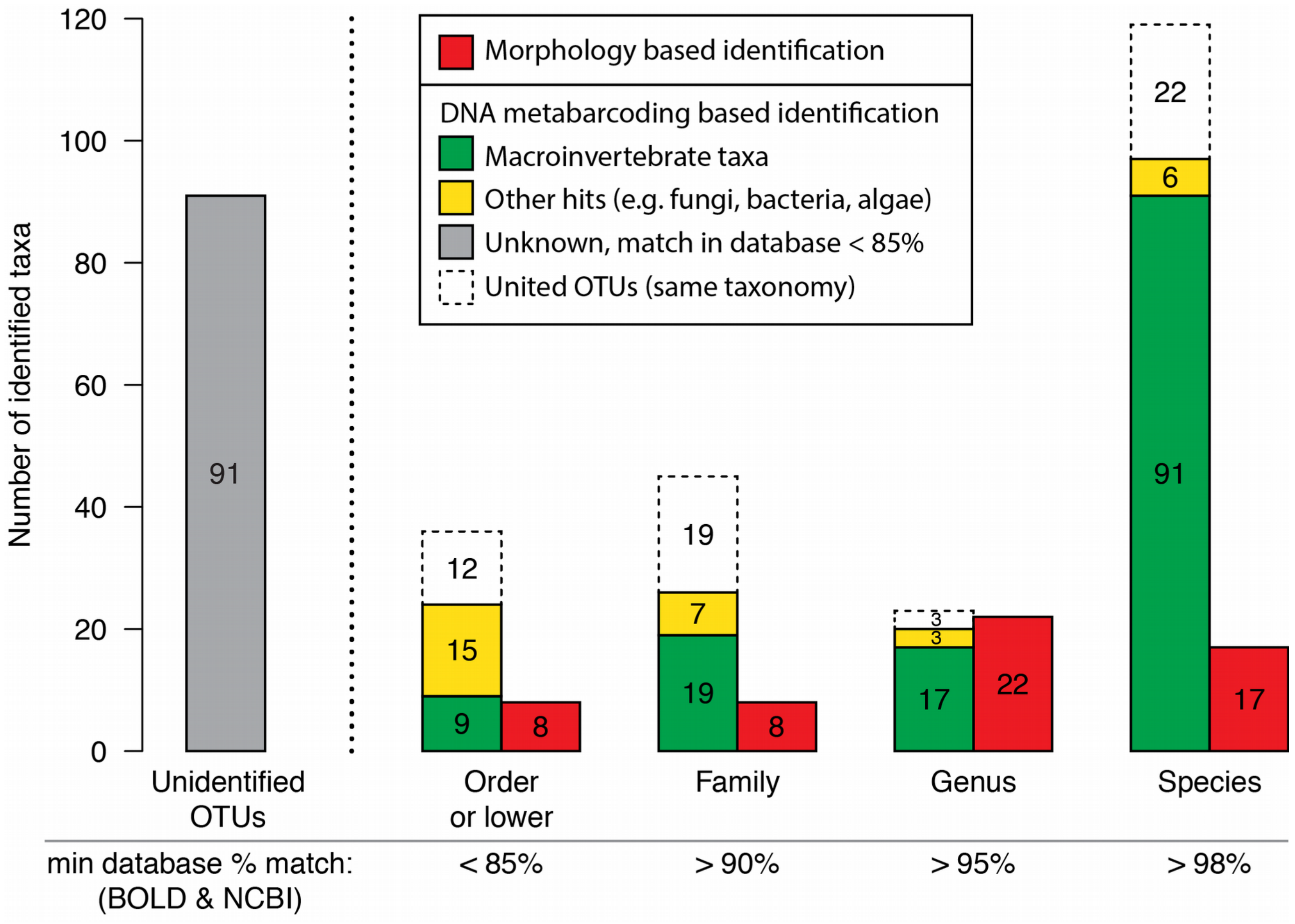

Figure S9: Comparison of macroinvertebrate taxa identified by DNA metabarcoding and morphological determination on different taxonomic levels. Below the bars the minimum required % match of the reference sequences required for sorting sequences into the respective category. OTUs with the same taxonomic hits in the reference database were merged into one single hit.
