# Supplementary material for: Sorting things out: Assessing effects of unequal specimen biomass on DNA metabarcoding
Source: Ecol Evol. 2017 Jul 28;7(17):6918–26. doi: 10.1002/ece3.3192 (PMC5587478; doi:10.1002/ece3.3192)
Supplement: Supplementary file 10 [file ECE3-7-6918-s010.pdf]

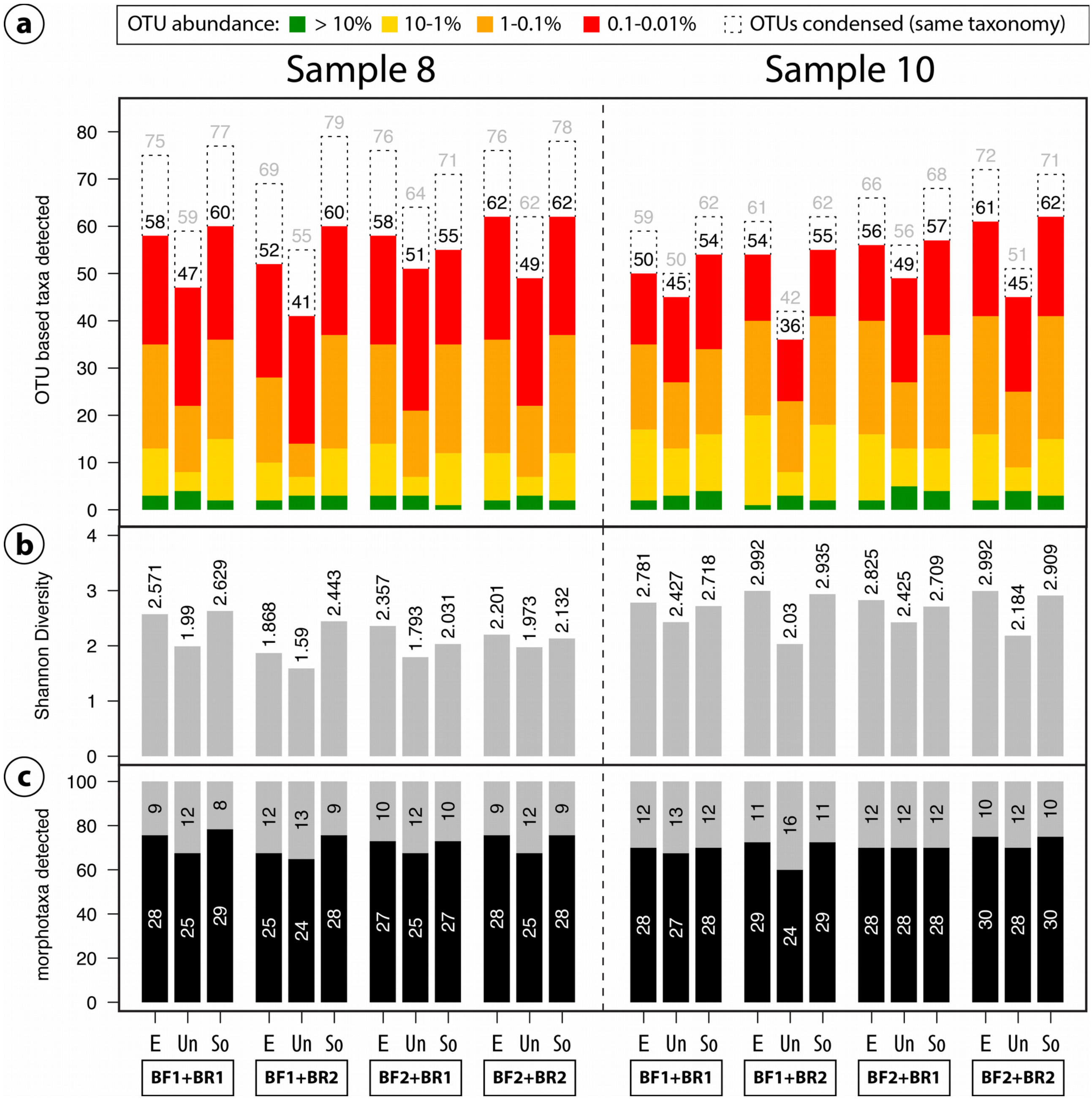

Figure S10: Comparison of macroinvertebrate taxa detection between unsorted (Un) and sorted (So) samples. Expected (E) taxa recovery was estimated by combining OTU abundances from S, M and L proportionately. **a)** Amount of OTUs and number of unique taxa identified with OTUs. OTU abundance for each taxon is indicated by color. **b)** Shannon Diversity for each sample. **c)** Proportion of morphologically identified taxa also detected with DNA metabarcoding is indicated by black bars.
